# Methyl 1-(4-fluoro­benz­yl)-1*H*-indazole-3-carboxyl­ate

**DOI:** 10.1107/S2414314623009951

**Published:** 2023-11-23

**Authors:** Takahiro Doi, Takayuki Sakai

**Affiliations:** a Osaka Institute of Public Health, Division of Hygienic Chemistry, Pharmaceutical Affairs Section, Nakamichi 1-3-3, Higashinari-ku, Osaka, 537-0025, Japan; Purdue University, USA

**Keywords:** crystal structure, synthetic cannabinoid, inter­mediate compound

## Abstract

The title compound, C_16_H_13_FN_2_O_2_, was synthesized by nucleophilic substitution of the indazole N—H hydrogen atom of methyl 1*H*-indazole-3-carboxyl­ate with 1-(bromo­meth­yl)-4-fluoro­benzene. In the crystal, some hydrogen-bond-like inter­actions are observed.

## Abstract 2

The title compound was synthesized by nucleophilic substitution of the indazole N—H hydrogen atom of methyl 1*H*-indazole-3-carboxyl­ate with 1-(bromo­meth­yl)-4-fluoro­benzene. In the crystal, some hydrogen-bond-like inter­actions are observed.

## Graphical abstract

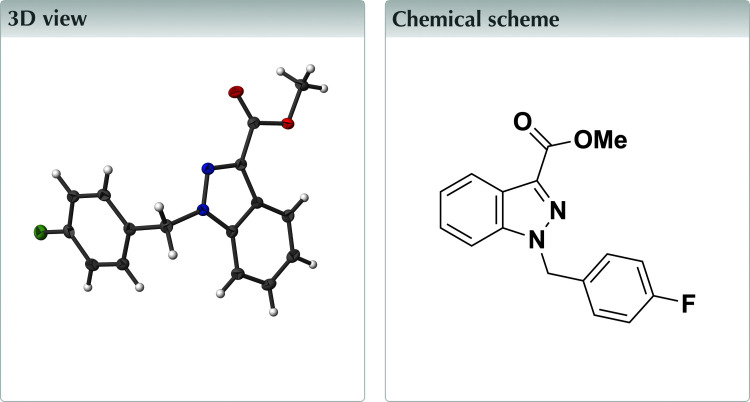

## Structure description

**Figure 1:**
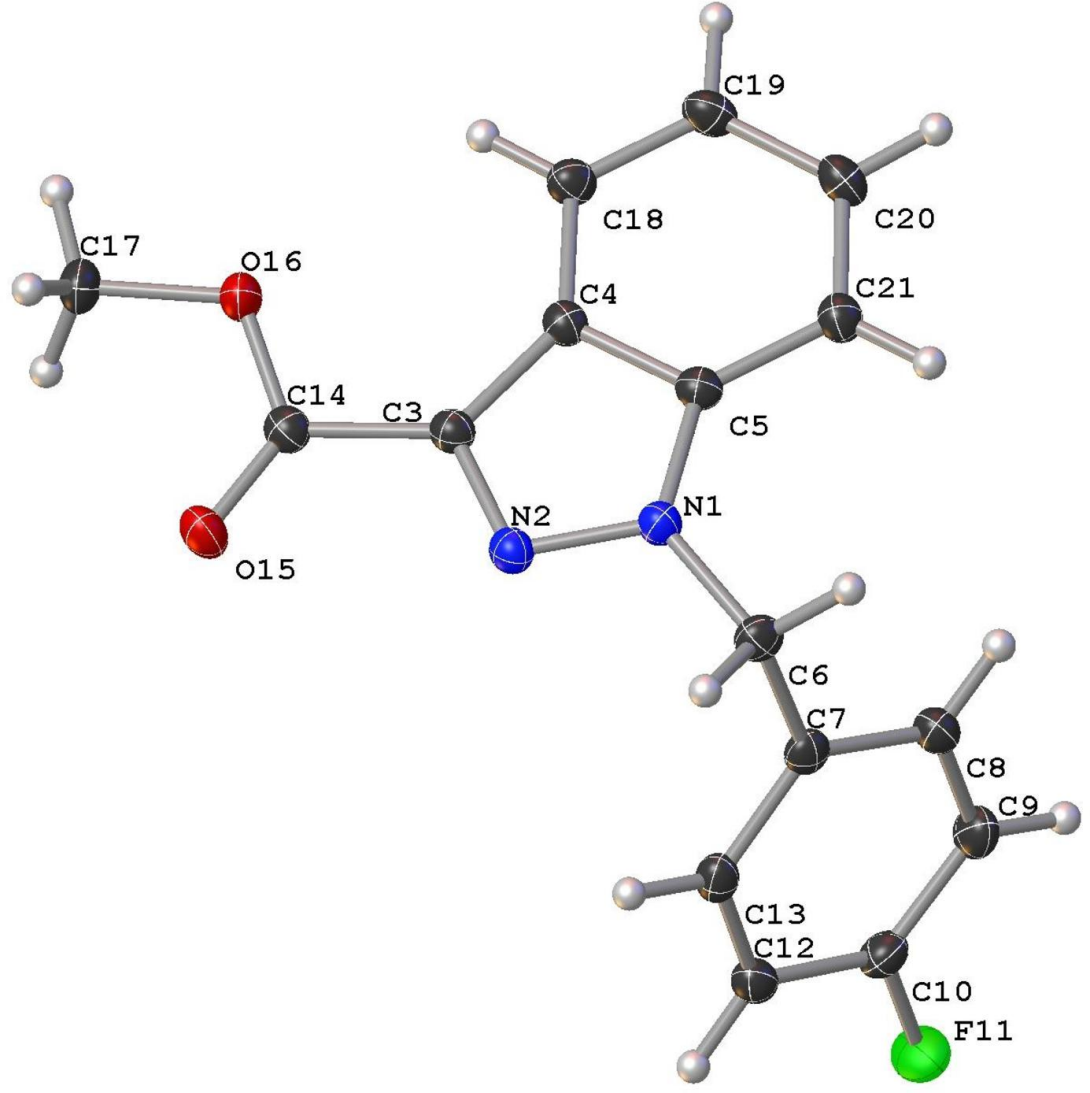
Mol­ecular structure of the title compound, with displacement ellipsoids drawn at the 50% probability level.

**Figure 2:**
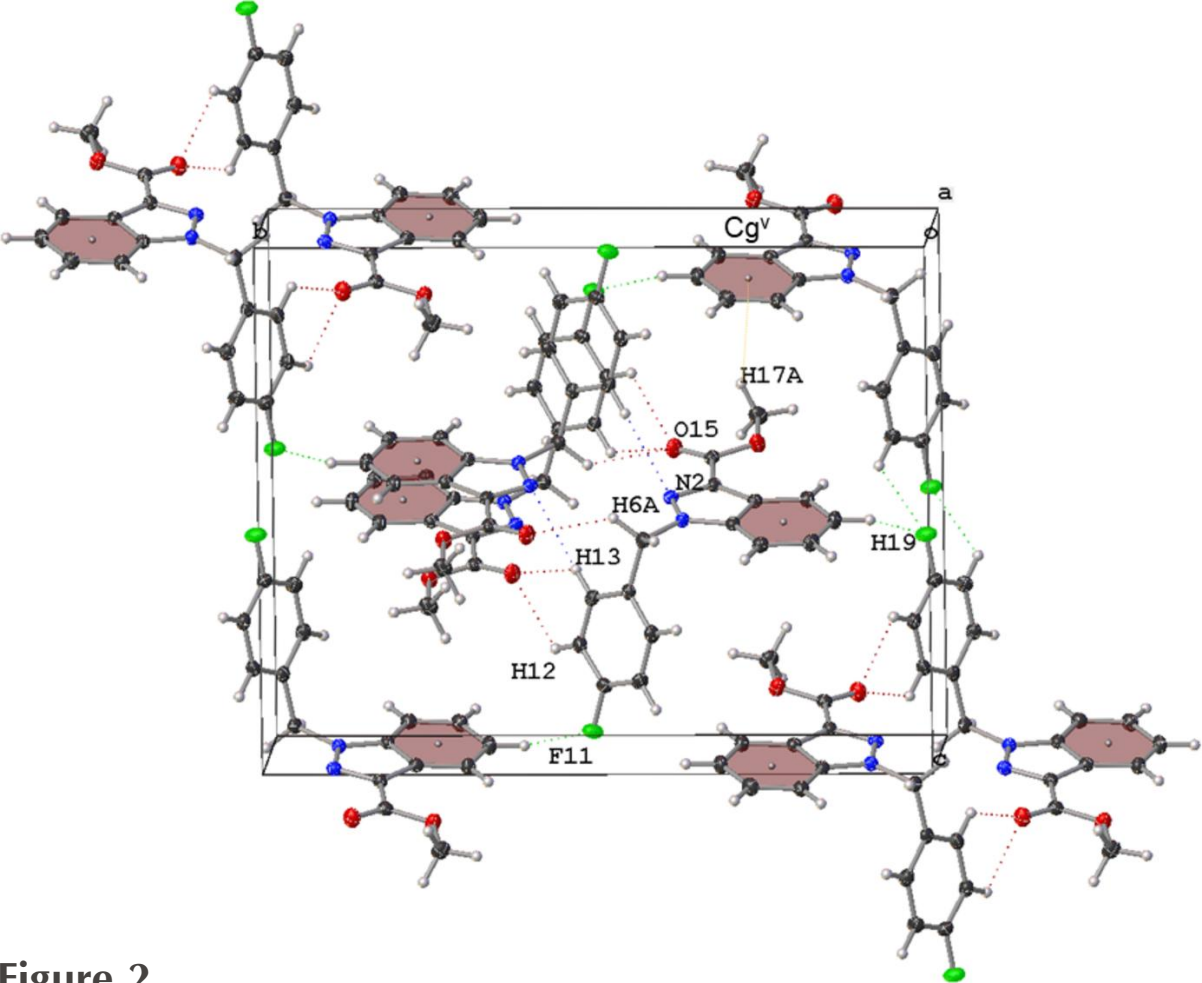
The crystal packing of the title compound.

Methyl 1-(4-fluoro­benz­yl)-1*H*-indazole-3-carboxyl­ate is an inter­mediate compound of synthetic cannabinoids, a class of compounds with a high potential for abuse as psychoactive substances, acting as the agonist of the cannabinoid type 1 receptor (Longworth *et al.*, 2017 ▸; Doi *et al.*, 2018 ▸; Cannaert *et al.*, 2020 ▸). The mol­ecule is composed of two planar segments connected at a bond angle of 110.90 (8)° at C6. The indazole ring is nearly coplanar with the ester moiety, suggesting that the ester moiety is conjugated with the aromatic ring. Furthermore, the C3—C14 bond distance is 1.4790 (14) Å, which provides further evidence for the existence of conjugation (Fig. 1 ▸). The crystal packing of the title compound is displayed in Fig. 2 ▸. At the centre of the crystal, two weak hydrogen-bond-like inter­actions (C13—H13⋯N2^ii^ and C6—H6*A*⋯O15^ii^) are formed between two adjacent mol­ecules related by inversion (Fig. 2 ▸) [symmetry operator: (ii) −*x*, −*y* + 1, −*z* + 1]. The hydrogen-donor mol­ecule also acts as acceptor of the same inter­actions, creating inversion-related dimers. In the extended structure, there are four more non-classical hydrogen-bond-like inter­actions and a weak C—H⋯π inter­action is also observed (Table 1 ▸).

## Synthesis and crystallization

The synthesis of methyl 1-(4-fluoro­benz­yl)-1*H*-indazole-3-carboxyl­ate was described previously (Doi *et al.*, 2018 ▸). In a microvial, the resulted compound was dissolved with ethyl acetate at a concentration of 3% (*w*/*v*). The microvial was left at room temperature for several months, resulting in the formation of several large rod shape crystals in the vial.

## Refinement

Crystal, data collection and refinement details are presented in Table 2 ▸.

## Supplementary Material

Crystal structure: contains datablock(s) I. DOI: 10.1107/S2414314623009951/zl4059sup1.cif

Structure factors: contains datablock(s) I. DOI: 10.1107/S2414314623009951/zl4059Isup4.hkl

Supporting information file. DOI: 10.1107/S2414314623009951/zl4059Isup3.cml

CCDC reference: 2308333

Additional supporting information:  crystallographic information; 3D view; checkCIF report

## Figures and Tables

**Table 1:** Hydrogen-bond geometry (Å, °) *Cg* is the centroid of the C4/C5/C18–C21 ring.

*D*—H⋯*A*	*D*—H	H⋯*A*	*D*⋯*A*	*D*—H⋯*A*
C6—H6*A*⋯O15^i^	0.99	2.67	3.3417 (13)	125
C12—H12⋯O15^ii^	0.95	2.61	3.2190 (12)	123
C13—H13⋯O15^ii^	0.95	2.62	3.2339 (12)	123
C13—H13⋯N2^i^	0.95	2.62	3.4578 (13)	148
C19—H19⋯F11^iii^	0.95	2.73	3.3840 (13)	127
C9—H9⋯F11^iv^	0.95	2.59	3.2577 (12)	127
C17—H17*A*⋯*Cg* ^v^	0.98	2.95	3.8114 (12)	148

Symmetry codes: (i) 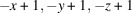 ; (ii) 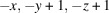 ; (iii) 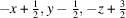 ; (iv) 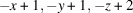 ; (v) 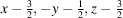 .

**Table 2:** Experimental details

Crystal data	
Chemical formula	C_16_H_13_FN_2_O_2_
*M*_r_	284.28
Crystal system, space group	Monoclinic, *P*2_1_/*n*
Temperature (K)	100
*a*, *b*, *c* (Å)	5.04322 (3), 18.11509 (13), 14.46487 (10)
β (°)	90.4600 (6)
*V* (Å^3^)	1321.45 (2)
*Z*	4
Radiation type	Cu *K*α
μ (mm^−1^)	0.88
Crystal size (mm)	0.32 × 0.13 × 0.12

Data collection	
Diffractometer	XtaLAB Synergy, Single source at home/near, HyPix-Arc 100
Absorption correction	Gaussian (*CrysAlis PRO*; Rigaku OD, 2023 ▸)
*T*_min_, *T*_max_	0.626, 1.000
No. of measured, independent and observed [*I* > 2σ(*I*)] reflections	47493, 2732, 2666
*R*_int_	0.035
(sin θ/λ)_max_ (Å^−1^)	0.627

Refinement	
*R*[*F* ^2^ > 2σ(*F* ^2^)], *wR*(*F* ^2^), *S*	0.031, 0.078, 1.03
No. of reflections	2732
No. of parameters	192
H-atom treatment	H-atom parameters constrained
Δρ_max_, Δρ_min_ (e Å^−3^)	0.30, −0.18

Computer programs: *CrysAlis PRO* (Rigaku OD, 2023 ▸), *SHELXT2018/2* (Sheldrick, 2015*a* ▸), *SHELXL2018/3* (Sheldrick, 2015*b* ▸), and *OLEX2* (Dolomanov *et al.*, 2009 ▸).

## full crystallographic data

### Crystal data

**Table:**

C_16_H_13_FN_2_O_2_	*F*(000) = 592
*M**_r_* = 284.28	*D*_x_ = 1.429 Mg m^−^^3^
Monoclinic, *P*2_1_/*n*	Cu *K*α radiation, λ = 1.54184 Å
*a* = 5.04322 (3) Å	Cell parameters from 29913 reflections
*b* = 18.11509 (13) Å	θ = 3.9–79.9°
*c* = 14.46487 (10) Å	µ = 0.88 mm^−^^1^
β = 90.4600 (6)°	*T* = 100 K
*V* = 1321.45 (2) Å^3^	Block, clear light colourless
*Z* = 4	0.32 × 0.13 × 0.12 mm

### Data collection

**Table:**

XtaLAB Synergy, Single source at home/near, HyPix-Arc 100 diffractometer	2732 independent reflections
Radiation source: fine-focus sealed X-ray tube, Enhance (Cu) X-ray Source	2666 reflections with *I* > 2σ(*I*)
Graphite monochromator	*R*_int_ = 0.035
Detector resolution: 10.0000 pixels mm^-1^	θ_max_ = 75.3°, θ_min_ = 3.9°
ω scans	*h* = −6→6
Absorption correction: gaussian (CrysAlisPro; Rigaku OD, 2023)	*k* = −21→22
*T*_min_ = 0.626, *T*_max_ = 1.000	*l* = −18→18
47493 measured reflections	

### Refinement

**Table:**

Refinement on *F*^2^	Hydrogen site location: inferred from neighbouring sites
Least-squares matrix: full	H-atom parameters constrained
*R*[*F*^2^ > 2σ(*F*^2^)] = 0.031	*w* = 1/[σ^2^(*F*_o_^2^) + (0.0369*P*)^2^ + 0.532*P*] where *P* = (*F*_o_^2^ + 2*F*_c_^2^)/3
*wR*(*F*^2^) = 0.078	(Δ/σ)_max_ = 0.001
*S* = 1.03	Δρ_max_ = 0.30 e Å^−^^3^
2732 reflections	Δρ_min_ = −0.18 e Å^−^^3^
192 parameters	Extinction correction: SHELXL-2018/3 (Sheldrick 2018), Fc^*^=kFc[1+0.001xFc^2^λ^3^/sin(2θ)]^-1/4^
0 restraints	Extinction coefficient: 0.0027 (3)
Primary atom site location: dual	

### Special details

**Table:**

Geometry. All esds (except the esd in the dihedral angle between two l.s. planes) are estimated using the full covariance matrix. The cell esds are taken into account individually in the estimation of esds in distances, angles and torsion angles; correlations between esds in cell parameters are only used when they are defined by crystal symmetry. An approximate (isotropic) treatment of cell esds is used for estimating esds involving l.s. planes.
Refinement. All the H atoms were included using a riding model starting from calculated positions (aromatic C—H = 0.95 Å, methylene C—H = 0.99 Å, and alkyl C—H = 1.00 Å). The *U*_iso_(H) values were fixed at 1.2 times the equivalent *U*_eq_ value of the parent C atoms (1.5 times for the methyl group).

### Fractional atomic coordinates and isotropic or equivalent isotropic displacement parameters (Å^2^)

**Table:**

	*x*	*y*	*z*	*U*_iso_*/*U*_eq_	
F11	0.20188 (14)	0.51087 (4)	0.93308 (4)	0.03075 (18)	
O16	0.11216 (15)	0.25606 (4)	0.38007 (5)	0.02287 (18)	
O15	0.03249 (15)	0.37833 (4)	0.38349 (5)	0.02512 (19)	
N1	0.65913 (16)	0.38193 (5)	0.56604 (6)	0.01720 (19)	
N2	0.45483 (16)	0.39395 (5)	0.50783 (6)	0.01800 (19)	
C5	0.71051 (19)	0.30814 (5)	0.57692 (7)	0.0168 (2)	
C4	0.52753 (19)	0.27022 (5)	0.52017 (7)	0.0170 (2)	
C3	0.37458 (19)	0.32774 (5)	0.47872 (7)	0.0172 (2)	
C7	0.63589 (19)	0.46374 (5)	0.70088 (7)	0.0180 (2)	
C6	0.7843 (2)	0.44440 (6)	0.61369 (7)	0.0194 (2)	
H6A	0.786848	0.487719	0.571991	0.023*	
H6B	0.970040	0.431674	0.629647	0.023*	
C12	0.2762 (2)	0.52949 (6)	0.77426 (7)	0.0201 (2)	
H12	0.131495	0.562982	0.771153	0.024*	
C13	0.4240 (2)	0.51310 (5)	0.69631 (7)	0.0191 (2)	
H13	0.380134	0.535846	0.639021	0.023*	
C14	0.1556 (2)	0.32536 (6)	0.41015 (7)	0.0185 (2)	
C18	0.5330 (2)	0.19243 (6)	0.51774 (7)	0.0200 (2)	
H18	0.411852	0.165588	0.480012	0.024*	
C21	0.9019 (2)	0.27162 (6)	0.63117 (7)	0.0198 (2)	
H21	1.025145	0.297972	0.668632	0.024*	
C10	0.3461 (2)	0.49563 (6)	0.85637 (7)	0.0212 (2)	
C20	0.9017 (2)	0.19570 (6)	0.62728 (8)	0.0226 (2)	
H20	1.028026	0.168985	0.663067	0.027*	
C8	0.7006 (2)	0.43131 (6)	0.78554 (7)	0.0219 (2)	
H8	0.845925	0.398046	0.789401	0.026*	
C19	0.7187 (2)	0.15640 (6)	0.57148 (8)	0.0225 (2)	
H19	0.723970	0.103984	0.571045	0.027*	
C9	0.5551 (2)	0.44705 (6)	0.86433 (7)	0.0242 (2)	
H9	0.598636	0.424934	0.922035	0.029*	
C17	−0.0982 (2)	0.24943 (6)	0.31188 (8)	0.0250 (2)	
H17A	−0.046710	0.275111	0.255159	0.038*	
H17B	−0.260822	0.271534	0.336094	0.038*	
H17C	−0.129430	0.197143	0.298222	0.038*	

### Atomic displacement parameters (Å^2^)

**Table:**

	*U*^11^	*U*^22^	*U*^33^	*U*^12^	*U*^13^	*U*^23^
F11	0.0376 (4)	0.0365 (4)	0.0183 (3)	0.0086 (3)	0.0041 (3)	−0.0015 (3)
O16	0.0232 (4)	0.0211 (4)	0.0241 (4)	0.0026 (3)	−0.0085 (3)	−0.0028 (3)
O15	0.0253 (4)	0.0218 (4)	0.0281 (4)	0.0045 (3)	−0.0065 (3)	0.0027 (3)
N1	0.0187 (4)	0.0158 (4)	0.0171 (4)	0.0004 (3)	−0.0013 (3)	0.0006 (3)
N2	0.0189 (4)	0.0187 (4)	0.0163 (4)	0.0013 (3)	−0.0001 (3)	0.0014 (3)
C5	0.0170 (4)	0.0165 (5)	0.0171 (5)	0.0003 (4)	0.0025 (4)	0.0006 (4)
C4	0.0160 (4)	0.0183 (5)	0.0168 (5)	0.0005 (4)	0.0012 (4)	0.0009 (4)
C3	0.0179 (5)	0.0169 (5)	0.0168 (5)	0.0008 (4)	0.0009 (4)	0.0009 (4)
C7	0.0190 (5)	0.0151 (5)	0.0199 (5)	−0.0037 (4)	−0.0022 (4)	−0.0014 (4)
C6	0.0202 (5)	0.0165 (5)	0.0216 (5)	−0.0027 (4)	−0.0004 (4)	−0.0010 (4)
C12	0.0209 (5)	0.0168 (5)	0.0224 (5)	0.0009 (4)	−0.0028 (4)	−0.0008 (4)
C13	0.0218 (5)	0.0166 (5)	0.0189 (5)	−0.0022 (4)	−0.0039 (4)	0.0018 (4)
C14	0.0184 (5)	0.0197 (5)	0.0172 (5)	0.0009 (4)	0.0011 (4)	0.0013 (4)
C18	0.0195 (5)	0.0174 (5)	0.0230 (5)	−0.0009 (4)	−0.0001 (4)	−0.0004 (4)
C21	0.0180 (5)	0.0216 (5)	0.0199 (5)	0.0007 (4)	−0.0015 (4)	0.0005 (4)
C10	0.0255 (5)	0.0208 (5)	0.0173 (5)	−0.0008 (4)	0.0003 (4)	−0.0034 (4)
C20	0.0202 (5)	0.0221 (5)	0.0255 (5)	0.0043 (4)	−0.0018 (4)	0.0042 (4)
C8	0.0236 (5)	0.0187 (5)	0.0235 (5)	0.0032 (4)	−0.0043 (4)	−0.0007 (4)
C19	0.0227 (5)	0.0162 (5)	0.0287 (5)	0.0019 (4)	0.0007 (4)	0.0019 (4)
C9	0.0315 (6)	0.0231 (5)	0.0179 (5)	0.0023 (4)	−0.0053 (4)	0.0019 (4)
C17	0.0231 (5)	0.0284 (6)	0.0234 (5)	0.0017 (4)	−0.0082 (4)	−0.0035 (4)

### Geometric parameters (Å, º)

**Table:**

F11—C10	1.3601 (12)	C12—H12	0.9500
O16—C14	1.3459 (13)	C12—C13	1.3887 (15)
O16—C17	1.4477 (12)	C12—C10	1.3801 (15)
O15—C14	1.2047 (13)	C13—H13	0.9500
N1—N2	1.3432 (12)	C18—H18	0.9500
N1—C5	1.3704 (13)	C18—C19	1.3767 (15)
N1—C6	1.4653 (13)	C21—H21	0.9500
N2—C3	1.3329 (13)	C21—C20	1.3765 (15)
C5—C4	1.4090 (14)	C10—C9	1.3770 (15)
C5—C21	1.4046 (14)	C20—H20	0.9500
C4—C3	1.4259 (13)	C20—C19	1.4135 (15)
C4—C18	1.4098 (14)	C8—H8	0.9500
C3—C14	1.4790 (14)	C8—C9	1.3900 (15)
C7—C6	1.5129 (14)	C19—H19	0.9500
C7—C13	1.3943 (14)	C9—H9	0.9500
C7—C8	1.3946 (14)	C17—H17A	0.9800
C6—H6A	0.9900	C17—H17B	0.9800
C6—H6B	0.9900	C17—H17C	0.9800
			
C14—O16—C17	114.47 (8)	O15—C14—O16	123.86 (9)
N2—N1—C5	111.93 (8)	O15—C14—C3	124.86 (9)
N2—N1—C6	119.68 (8)	C4—C18—H18	120.9
C5—N1—C6	128.23 (8)	C19—C18—C4	118.24 (9)
C3—N2—N1	106.36 (8)	C19—C18—H18	120.9
N1—C5—C4	106.64 (8)	C5—C21—H21	121.7
N1—C5—C21	130.70 (9)	C20—C21—C5	116.59 (10)
C21—C5—C4	122.66 (9)	C20—C21—H21	121.7
C5—C4—C3	103.78 (8)	F11—C10—C12	118.50 (9)
C5—C4—C18	119.27 (9)	F11—C10—C9	118.41 (9)
C18—C4—C3	136.95 (9)	C9—C10—C12	123.09 (10)
N2—C3—C4	111.28 (9)	C21—C20—H20	119.1
N2—C3—C14	117.47 (9)	C21—C20—C19	121.78 (10)
C4—C3—C14	131.23 (9)	C19—C20—H20	119.1
C13—C7—C6	119.54 (9)	C7—C8—H8	119.6
C13—C7—C8	119.06 (9)	C9—C8—C7	120.80 (10)
C8—C7—C6	121.36 (9)	C9—C8—H8	119.6
N1—C6—C7	110.90 (8)	C18—C19—C20	121.45 (10)
N1—C6—H6A	109.5	C18—C19—H19	119.3
N1—C6—H6B	109.5	C20—C19—H19	119.3
C7—C6—H6A	109.5	C10—C9—C8	118.12 (10)
C7—C6—H6B	109.5	C10—C9—H9	120.9
H6A—C6—H6B	108.0	C8—C9—H9	120.9
C13—C12—H12	121.0	O16—C17—H17A	109.5
C10—C12—H12	121.0	O16—C17—H17B	109.5
C10—C12—C13	117.95 (9)	O16—C17—H17C	109.5
C7—C13—H13	119.5	H17A—C17—H17B	109.5
C12—C13—C7	120.97 (9)	H17A—C17—H17C	109.5
C12—C13—H13	119.5	H17B—C17—H17C	109.5
O16—C14—C3	111.28 (8)		

### Hydrogen-bond geometry (Å, º)

*Cg* is the centroid of the C4/C5/C18–C21 ring.

**Table:**

*D*—H···*A*	*D*—H	H···*A*	*D*···*A*	*D*—H···*A*
C6—H6*A*···O15^i^	0.99	2.67	3.3417 (13)	125
C12—H12···O15^ii^	0.95	2.61	3.2190 (12)	123
C13—H13···O15^ii^	0.95	2.62	3.2339 (12)	123
C13—H13···N2^i^	0.95	2.62	3.4578 (13)	148
C19—H19···F11^iii^	0.95	2.73	3.3840 (13)	127
C9—H9···F11^iv^	0.95	2.59	3.2577 (12)	127
C17—H17*A*···*Cg*^v^	0.98	2.95	3.8114 (12)	148

Symmetry codes: (i) −*x*+1, −*y*+1, −*z*+1; (ii) −*x*, −*y*+1, −*z*+1; (iii) −*x*+1/2, *y*−1/2, −*z*+3/2; (iv) −*x*+1, −*y*+1, −*z*+2; (v) *x*−3/2, −*y*−1/2, *z*−3/2.
